# NCX1 and NCX3 as potential factors contributing to neurodegeneration and neuroinflammation in the A53T transgenic mouse model of Parkinson’s Disease

**DOI:** 10.1038/s41419-018-0775-7

**Published:** 2018-06-25

**Authors:** Rossana Sirabella, Maria Josè Sisalli, Giulia Costa, Katia Omura, Gaetano Ianniello, Annalisa Pinna, Micaela Morelli, Gianfranco Maria Di Renzo, Lucio Annunziato, Antonella Scorziello

**Affiliations:** 1 Division of Pharmacology, Department of Neuroscience, School of Medicine, Federico II University of Naples, Naples, Italy; 2 Department of Biomedical Sciences, Section of Neuropsychopharmacology, University of Cagliari, Cagliari, Italy; 3 National Research Council of Italy (CNR), Institute of Neuroscience, Cagliari, Italy; 4 IRCCS SDN, Naples, Italy

## Abstract

Na^+^-Ca^2+^ exchanger (NCX) isoforms constitute the major cellular Ca^2+^ extruding system in neurons and microglia. We herein investigated the role of NCX isoforms in the pathophysiology of Parkinson’s disease (PD). Their expression and activity were evaluated in neurons and glia of mice expressing the human A53T variant of α-synuclein (A53T mice), an animal model mimicking a familial form of PD. Western blotting revealed that NCX3 expression in the midbrain of 12-month old A53T mice was lower than that of wild type (WT). Conversely, NCX1 expression increased in the striatum. Immunohistochemical studies showed that glial fibrillary acidic protein (GFAP)-positive astroglial cells significantly increased in the substantia nigra *pars compacta* (SNc) and in the striatum. However, the number and the density of tyrosine hydroxylase (TH)-positive neurons decreased in both brain regions. Interestingly, ionized calcium binding adaptor molecule 1 (IBA-1)-positive microglial cells increased only in the striatum of A53T mice compared to WT. Double immunostaining studies showed that in A53T mice, NCX1 was exclusively co-expressed in IBA-1-positive microglial cells in the striatum, whereas NCX3 was solely co-expressed in TH-positive neurons in SNc. Beam walking and pole tests revealed a reduction in motor performance for A53T mice compared to WT. In vitro experiments in midbrain neurons from A53T and WT mice demonstrated a reduction in NCX3 expression, which was accompanied by mitochondrial overload of Ca^2+^ ions, monitored with confocal microscopy by X-Rhod-1 fluorescent dye. Collectively, in vivo and in vitro findings suggest that the reduction in NCX3 expression and activity in A53T neurons from midbrain may cause mitochondrial dysfunction and neuronal death in this brain area, whereas NCX1 overexpression in microglial cells may promote their proliferation in the striatum.

## Introduction

Parkinson’s disease (PD) is characterized by a progressive loss of dopaminergic neurons in the substantia nigra *pars compacta* (SNc)^1,2^. The clinical hallmarks of the disease are bradykinesia, hypokinesia, resting tremor, rigidity, and postural instability^3^.

In addition to dopaminergic degeneration in the SNc, PD is neuropathologically characterized by the presence of Lewy bodies and intracytoplasmic eosinophilic inclusions in injured or fragmented neurons^4^, with α-synuclein as the major fibrillary component^5^. The exact mechanism underlying selective mesostriatal dopaminergic neurodegeneration is still unclear. However, many cellular mechanisms are known to be involved in the pathogenesis of PD, including oxidative stress^6^, intracellular Ca^2+^ homeostasis impairment^7^, and mitochondrial dysfunction^8^. Clinical, epidemiological, and experimental evidence has pinpointed neuroinflammation as a major driver of disease progression and glial cell activation as a key player in dopaminergic neuronal degeneration^9^. Indeed, studies of the SNc from patients with PD and of 1-methyl-4-phenyl-1,2,3,6-tetrahydropyridine (MPTP)-treated mice show an infiltration of CD4+ lymphocytes^10^, whereas other studies of the SNc from PD patients and of individuals intoxicated with MPTP have reported gliosis^11,12^. Interestingly, a study performed in monkeys overexpressing the A53T variant of α-synuclein^13^ shows that dopaminergic neuronal degeneration is associated with long-term microgliosis in the midbrain—a finding of particular relevance since it correlates misfolded α-synuclein with glial activation.

Although most cases of PD occur sporadically, mutations in several genes have been linked to the genetic forms of PD. For instance, mutations in parkin (PARK2), DJ-1 (PARK7), and PINK1 (PARK6) are associated with recessive early-onset forms of PD, whereas mutations in α-synuclein (PARK1–4) and LRRK2 (PARK8) are responsible for the dominant forms of familial PD. The activity and cellular distribution of the proteins encoded by these genes have not been completely elucidated. However, recent findings have led scientists to hypothesize their possible involvement in the regulation of mitochondrial morphology and function, as well as in cellular metabolism. Indeed, PINK1 and parkin play crucial roles in the mitochondrial dynamics and function in PD^14^, whereas mutations in DJ-1 and parkin render animals more susceptible to oxidative stress^15,16^. Moreover, dysfunction in PINK1 causes mitochondrial Ca^2+^ overload due to the inhibition of the mitochondrial Na^+^/Ca^2+^ exchanger^14^, thus confirming that mitochondrial dysfunction might play an important role in the pathogenesis of neuronal damage in PD. This hypothesis has been further supported by the localization of α-synuclein on mitochondria, where it contributes to impairment of respiratory complex I activity, oxidative modification of mitochondrial proteins, and increased mitochondrial Ca^2+^ concentrations ([Ca^2+^]_m_)^17–19^.

Interestingly, α-synuclein, through its ability to modulate brain lipid metabolism, is also involved in cerebral inflammatory responses^20^. Indeed, α-synuclein mutant forms, including A53T, stimulate the production of proinflammatory cytokines and the activation of microglia through an increase in phosphatidic acid (PtdOH) formation^20^. These findings, which correlate with mitochondrial dysfunction in neurons^21,22^, suggest that mitochondria might indeed play a role in PD-associated neuroinflammation by releasing factors able to activate proinflammatory cytokines^23,24^.

Our recent studies have demonstrated that among the three isoforms of the plasmamembrane Na^+^/Ca^2+^ exchanger (NCX), NCX1 plays a crucial role in the regulation of microglia activation in ischemic rat brain through a Ca^2+^-dependent mechanism^25^. Instead, NCX3, besides being expressed at the plasmamembrane level, is also localized on the outer mitochondrial membrane where it regulates mitochondrial Ca^2+^ efflux under physiological and pathological conditions^26,27^.

Furthermore, a study performed on human dopaminergic neurons has shown that plasmalemmal NCX2 and NCX3 may contribute to mitochondrial Na^+^ and Ca^2+^ exchange by acting downstream of PINK1, thereby preventing neurodegeneration due to [Ca^2+^]_m_ accumulation^28^. In addition, NCX1 gives rise to several splicing variants that appear to be selectively expressed in different regions and cellular populations of the brain. In fact, NCX1 mRNA can be detected in the midbrain, where dopaminergic cell bodies are localized^29^, and in the striatum, where the terminal projections of dopaminergic nigrostriatal neurons are found^30^.

In light of these premises and the fact that Ca^2+^ signaling is relevant to promote microglial cells activation^25,31,32^, we explored whether changes in the expression of the three NCX isoforms in glial and neuronal cells might play a role in the pathophysiology of PD. Building on this hypothesis, we thus set out to investigate whether mitochondrial dysfunction is accountable for the activation of neuroinflammation, dopaminergic neuronal degeneration, and motor impairment in 12-month-old mice expressing the human A53T variant of α-synuclein.

## Results

### NCX1 increases in striatum, whereas NCX3 decreases in midbrain of 12-month-old A53T mice

**Fig. 1:**
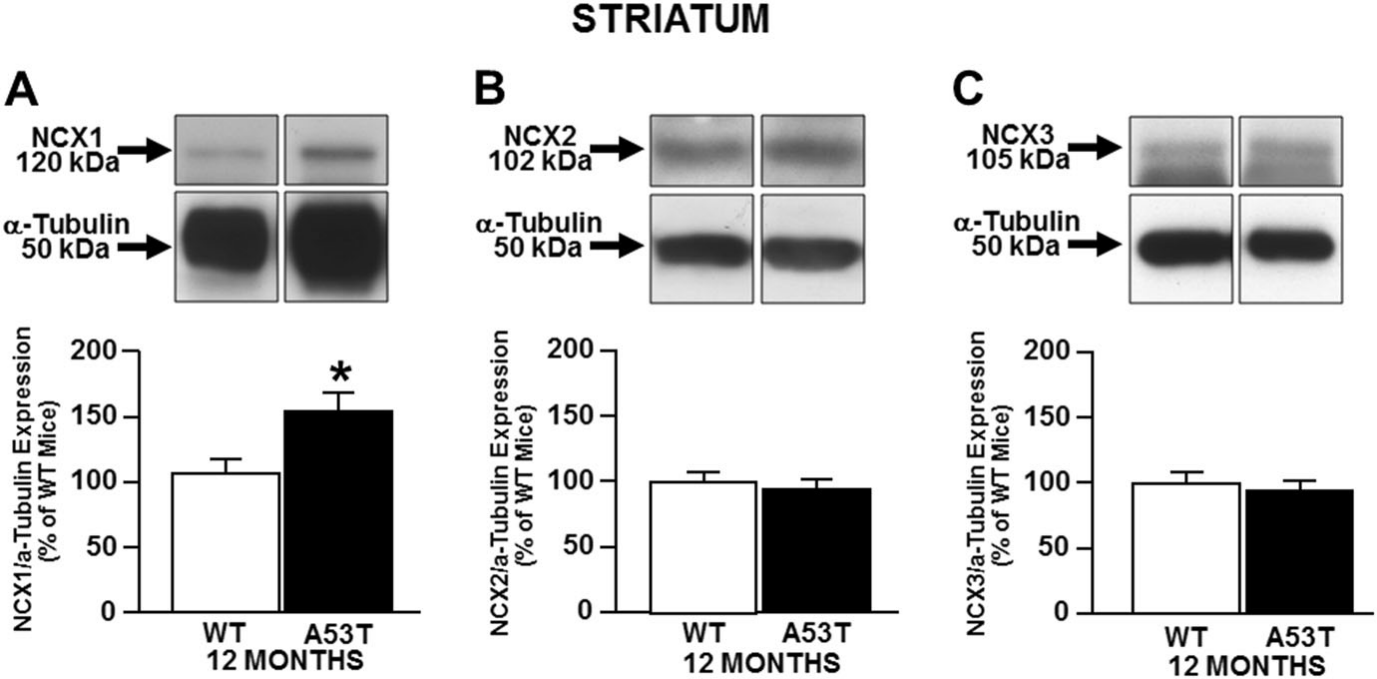
NCXs expression in the striatum of A53T and WT mice. The bar graphs report the mean ± S.E.M. of the percentage of NCX1 (**a**), NCX2 (**b**), and NCX3 (**c**) expression obtained in striatum of A53T and WT mice and normalized to the respective α-Tubulin. *N* = 8 animals were used for each experimental group. *P < 0.05 vs respective WT mice

**Fig. 2:**
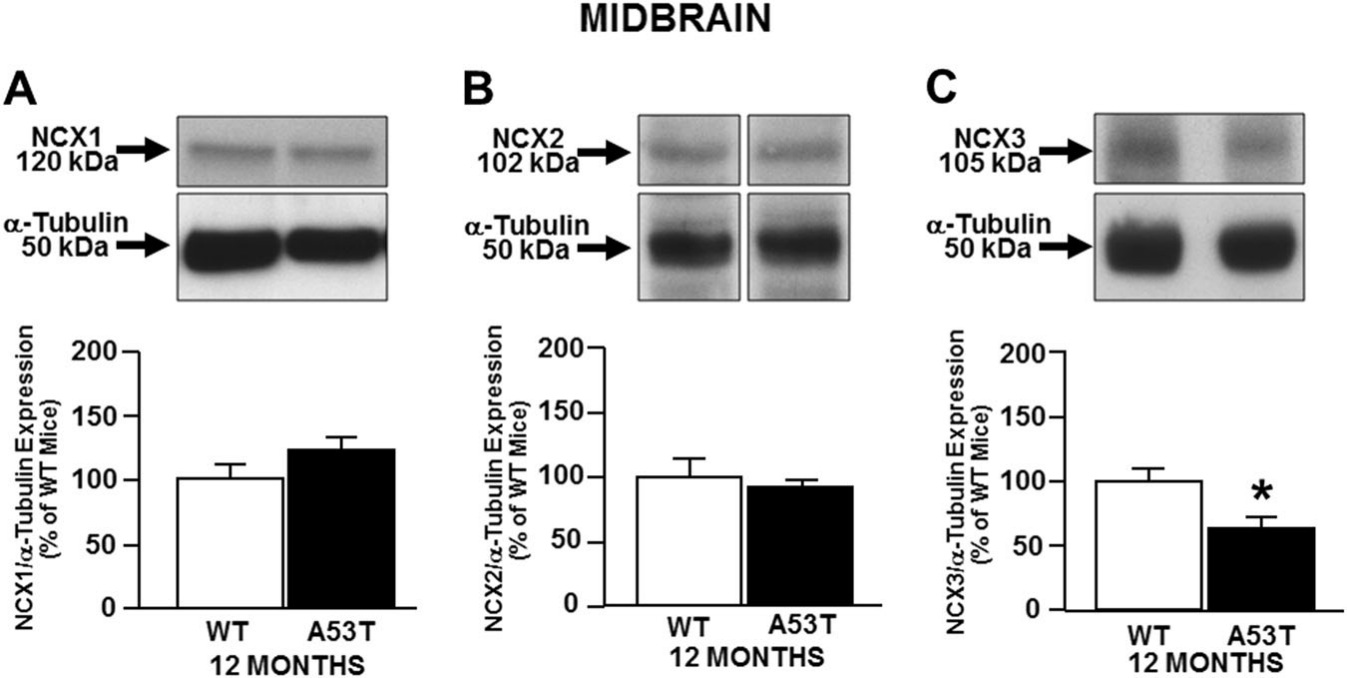
NCXs expression in midbrain of A53T and WT mice. The bar graphs report the mean ± S.E.M. of the percentage of NCX1 (**a**), NCX2 (**b**), and NCX3 (**c**) expression obtained in midbrain of A53T and WT mice and normalized to the respective α-Tubulin. *N* = 8 animals were used for each experimental group. *P < 0.05 vs respective WT mice

To understand the role of the Na^+^-Ca^2+^ exchanger (NCX) isoforms in the pathophysiology of PD, we performed Western blot experiments in A53T and WT mice, the former being a mouse model of a familial form of the disease. We found that NCX1 levels were increased in the striatum of A53T mice (Fig. 1a), whereas NCX2 and NCX3 levels were similar to those of WT (Fig. 1b, c). Conversely, in the midbrain of A53T mice, NCX3 levels were lower than those of WT, whereas NCX1 and NCX2 were the same as those of WT (Fig. 2).

### GFAP-positive astrocytes increase in striatum and in SNc, whereas IBA-1-positive microglial cells rise only in striatum of 12-month old A53T mice

**Fig. 3:**
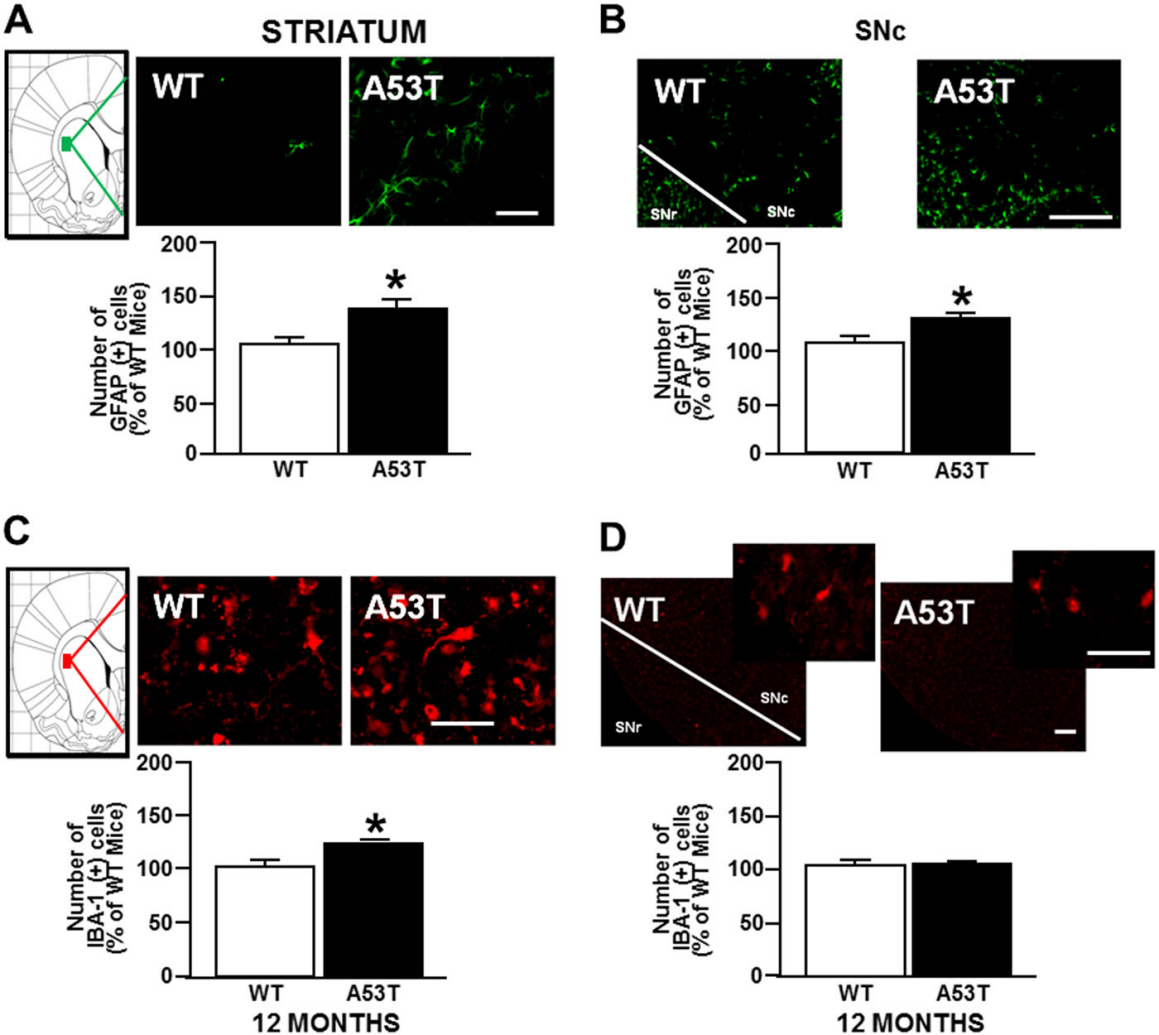
Immunoreactivity for GFAP and IBA-1 in striatum and substantia nigra *pars compacta* (SNc) of A53T and WT mice. Representative sections and histograms of striatum and SNc immunostained for GFAP and IBA-1. The bar graphs report the number of GFAP-positive cells (green) in striatum (**a**) and SNc (**b**) of A53T mice, expressed as a percentage of WT mice. **c** The bar graphs report the number of IBA-1-positive cells (red) in striatum and **d** in SNc of 12-month-old A53T mice, expressed as a percentage of WT mice. *N* = 8 animals were used for each experimental group. *P < 0.05 vs respective WT mice. Scale bar: 50, 75, and 100 µm

Immunohistochemistry experiments performed in A53T and WT mice demonstrated an increase in the number of glial fibrillary acidic protein (GFAP)-positive astrocytes in both the striatum (Fig. 3a) and the SNc (Fig. 3b) compared with WT mice. Interestingly, an increase in the number of ionized calcium binding adaptor molecule 1 (IBA-1)-positive microglial cells was observed in the striatum (Fig. 3c) but not in the SNc (Fig. 3d) compared with WT mice.

These changes were accompanied by a significant reduction in the number and density of tyrosine hydroxylase (TH)-positive neurons in the SNc (Figure S1B), and by a decrease in the density of TH-positive fibers in the striatum of A53T mice compared with WT mice (Figure S1A). No change in the volume of the SNc was observed.

### NCX1 isoform is co-expressed with IBA-1-positive microglial cells in striatum, whereas NCX3 isoform is co-expressed in TH-positive neurons in SNc of A53T mice

**Fig. 4:**
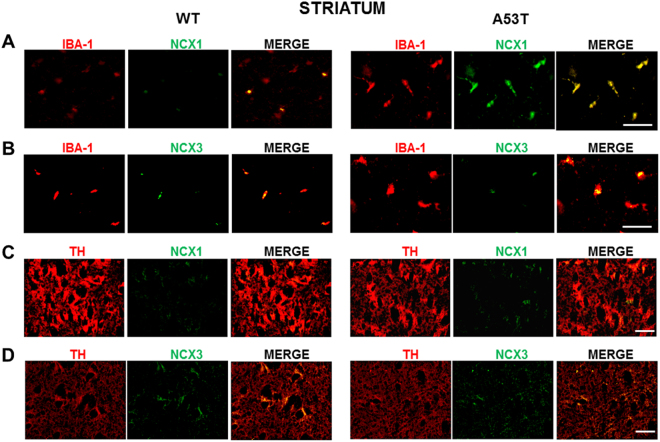
Double immunoreactivity for IBA-1 and NCX1 or NCX3 and for TH and NCX1 or NCX3 in striatum of A53T mice. **a** Representative sections of striatum immunostained for IBA-1 and NCX1. **b** Representative sections of striatum immunostained for IBA-1 and NCX3. **c** Representative sections of striatum immunostained for TH and NCX1. **d** Representative sections of striatum immunostained for TH and NCX3. *N* = 8 animals were used for each experimental group. Scale bar: 50 and 100 µm

**Fig. 5:**
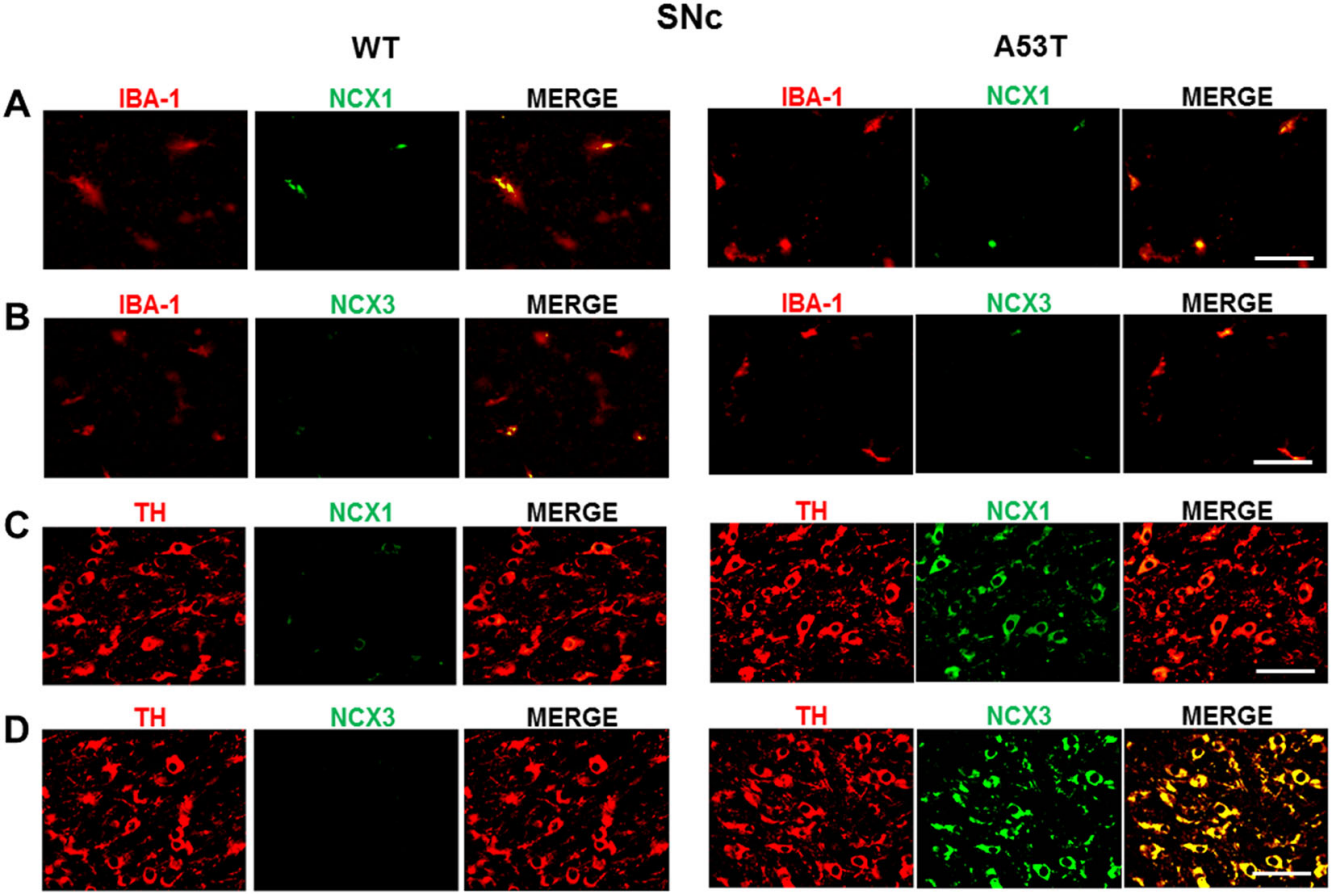
Double immunoreactivity for IBA-1 and NCX1 or NCX3 and for TH and NCX1 or NCX3 in substantia nigra *pars compacta* (SNc) of A53T mice. **a** Representative sections of SNc immunostained for IBA-1 and NCX1. **b** Representative sections of SNc immunostained for IBA-1 and NCX3. **c** Representative sections of SNc immunostained for TH and NCX1. **d** Representative sections of SNc immunostained for TH and NCX3. *N* = 8 animals were used for each experimental group. Scale bar: 50 and 100 µm

Microglial cells and dopaminergic neurons were immunostained for IBA-1+NCX1, IBA-1+NCX3, TH+NCX1, and TH+NCX3 in the striatum and SNc of A53T and WT mice. The results reported in Figs. 4 and 5, S2 and S3 demonstrated that IBA-1+NCX3 and TH+NCX1 did not significantly overlap in the striatum and SNc of A53T mice compared with WT mice. However, NCX1 immunostaining (green) significantly overlapped with IBA-1-labeled microglial cells (red) in the striatum (Fig. 4 and S2) but not in SNc of A53T mice, as compared with WT mice. Moreover, NCX3 immunostaining (green) significantly overlapped with TH-labeled dopaminergic neurons (red) in the SNc but not in the striatum of A53T mice (Fig. 5 and S3).

### Motor performance impairment in A53T mice

Motor ability in A53T mice showed a general tendency to worsen compared to WT mice. Indeed, as revealed by the pole test, which evaluates imbalance and bradykinesia, A53T mice spent more time climbing down the pole than did WT mice (Figure S4A). Similarly, the motor performance and the coordination of A53T mice, evaluated by measuring the mean scores of the beam walking test, was also impaired. Indeed, it took these mice much longer than WT to walk across the beam, whereas the number of steps and the errors per step were similar in both groups (Figure S4C).

Finally, the open field test, which measures spontaneous exploratory locomotor activity, revealed a significant impairment in the exploratory behavior of A53T mice, compared to WT mice (Figure S4B).

### NCX3 protein expression was decreased, whereas cytosolic and mitochondrial calcium concentrations were increased in midbrain neurons from A53T mice

**Fig. 6:**
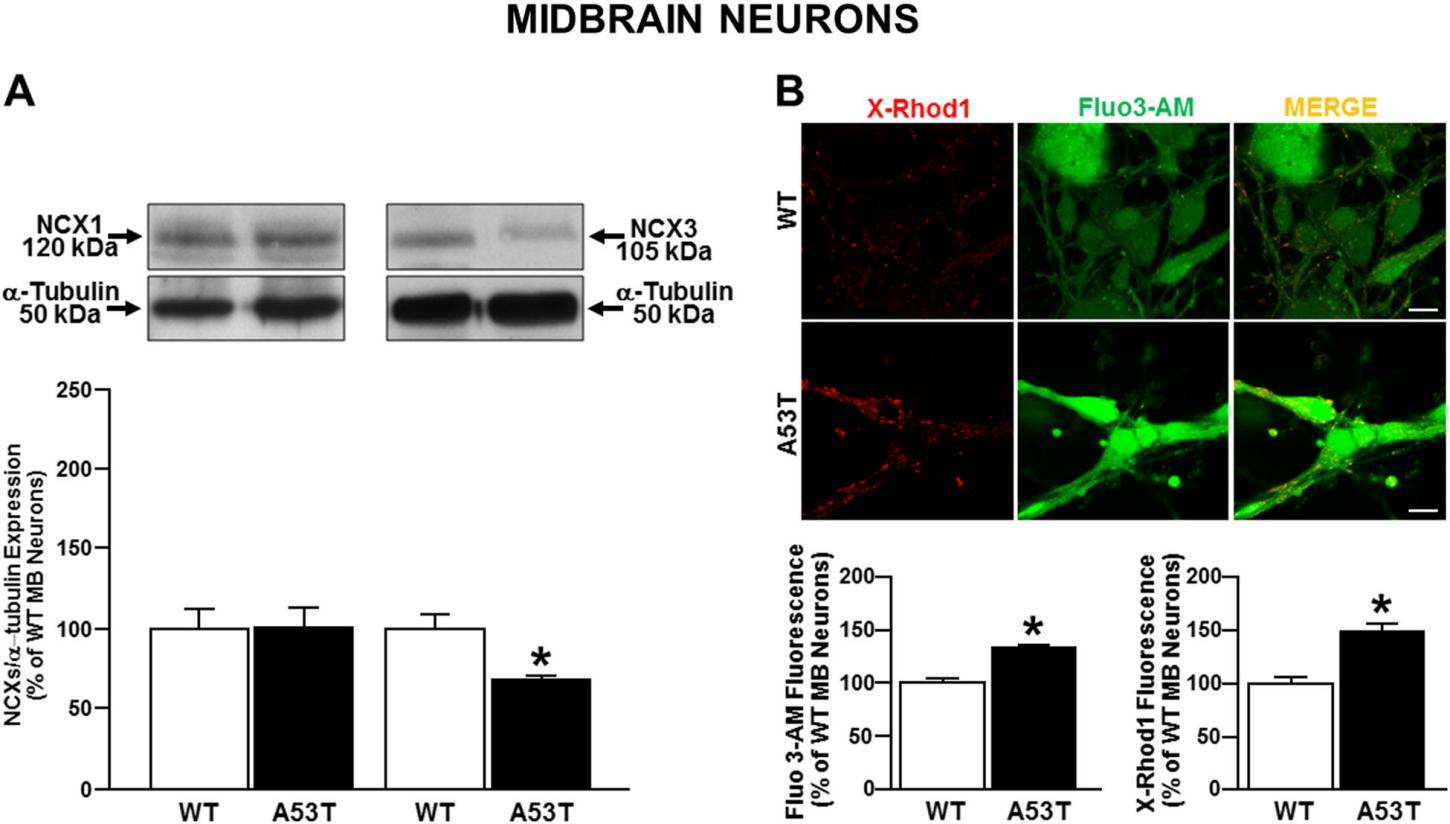
NCX expression and activity in midbrain neurons obtained from A53T mouse embryos. **a** Bar graphs report the mean ± S.E.M. of the densitometric values of NCX1 and NCX3 expression in primary midbrain A53T and WT neurons normalized to the respective α-Tubulin. *P < 0.05 vs respective WT neurons; **b** cytosolic and mitochondrial Ca^2+^ concentrations in midbrain neurons obtained from A53T and WT mouse embryos. Values were expressed as mean of the percentage ± S.E.M. *P < 0.05 vs WT neurons. Images are representative of X-Rod 1 (red) and Fluo-3 (green) distribution in cytosol and in mitochondria in WT and in A53T Transgenic neurons respectively. Scale bar: 100 μm

Experiments performed in primary mature midbrain neurons (10DIV) from A53T and WT mice embryos confirmed that NCX3 protein levels were reduced in A53T neurons as compared to WT neurons, whereas no differences in NCX1 protein levels were detected (Fig. 6a). Finally, functional experiments performed in A53T neurons demonstrated an increase in [Ca^2+^]_c_ and in [Ca^2+^]_m_ in comparison to WT neurons (Fig. 6b).

## Discussion

In the present study, we have demonstrated that in transgenic mice bearing the α-synuclein A53T human mutation the three NCX isoforms are differently modulated. Indeed, in the midbrain of A53T mice, NCX3 was reduced, whereas NCX1 and NCX2 were unaltered, compared to WT mice. Conversely, in the striatum, NCX1 protein levels were increased. Interestingly, NCX1 was co-expressed with IBA-1-positive cells. Such evidence suggests that the increase in NCX1 expression observed in the striatum of A53T mice might be ascribed to the occurrence of microgliosis in this brain area. This result is in line with those reported by Boscia et al.^25^ who demonstrated that in the ischemic brain NCX1 is overexpressed in microglial cells invading the ischemic core.

Recently, accumulating evidence suggests that reactive glial cells, by virtue of their inflammatory properties, may play a significant role in the cascade of events prompting and sustaining neuronal death in PD^33,34^. Therefore, we hypothesize that the increase in NCX1 protein expression might play a detrimental role in PD progression by promoting microglial activation, as already suggested by Ago and co-workers^35^. These authors indeed demonstrated that treating MPTP-exposed mice with SEA0400, an NCX1 inhibitor, ameliorates motor activity and reduces dopaminergic neuronal loss possibly by inhibiting NCX-mediated Ca^2+^ influx, a phenomenon leading to ERK phosphorylation and lipid peroxidation. Remarkably, the finding reported in the present study that high levels of NCX1 are detected in IBA-1-positive microglial cells in the striatum of A53T mice, a brain area containing abnormal deposition of α-synuclein, provides new insights into the mechanism underlying the detrimental implications of NCX1 activation in the pathophysiology of PD. Moreover, we also observed a reduction in NCX3 expression in dopaminergic neurons in the SNc of A53T mice, a phenomenon that was accompanied by an increase in mitochondrial Ca^2+^ concentrations and neuronal death. Such evidence led us to speculate on another possible mechanism contributing to neuronal degeneration. In particular, we hypothesize that changes in NCX1 and NCX3 protein expression and activity, in two core regions of the dopaminergic nigrostriatal circuit, albeit in different cellular population, might perturb intracellular Ca^2+^ concentration, which will eventually lead to neuronal loss in PD. This is in line with the recently proposed hypothesis that the selective degeneration of neurons in the SNc might be correlated with an elevated intracellular and mitochondrial Ca^2+^ rise^36–39^, which in turn promotes mitochondrial damage^19^ and neuroinflammation^31,32^. Therefore, the results reported in the present study corroborate the hypothesis that alterations in Ca^2+^ homeostasis might contribute to the pathophysiology of PD^3,40–43^ by setting in motion a marked inflammatory response in the brain.

The most intriguing aspect of the present study resides in the identification of NCX1 and NCX3 as players in the intracellular mechanisms leading to neuronal degeneration. Indeed, we speculate that the increase in [Ca^2+^]_c_ and [Ca^2+^]_m_ observed in A53T midbrain neurons might be a consequence of NCX3 impairment. This result is in accordance with those of previous studies recently demonstrating the localization of NCX3 on the outer mitochondrial membrane, where it contributes to mitochondrial Ca^2+^ extrusion^26^. This finding strongly supports the hypothesis that in the A53T model of PD a causal relationship between perturbation of intracellular Ca^2+^ homeostasis, mitochondrial membrane potential and mitochondrial dysfunction^19^ leads to neuronal degeneration. Consistently, increasing evidence indicates that NCX3, which is highly expressed in the brain, plays a pivotal role in the maintenance of intracellular Na^+^ and Ca^2+^ homeostasis in brain ischemia and in neurodegenerative diseases, thus mediating neuroprotective effects^27,44–49^. Indeed, deletion of the NCX3 gene in mice has detrimental consequences on basal synaptic transmission, long-term potentiation, spatial learning, and memory performance^50^.

Therefore, the reduction in NCX3 expression and activity observed in A53T-derived midbrain neurons as a consequence of abnormal α-synuclein deposition, determines an alteration of intracellular calcium homeostasis, whose levels are primarily regulated by the exchanger^44,47^, accompanied to mitochondrial dysfunction. These results are suggestive of the hypothesis that the deregulation of calcium homeostasis might led to the impairment of dopaminergic neurons observed in A53T transgenic mice. These effects might in turn stimulate proinflammatory factors release that prompt NCX1-mediated microglial activation in striatum. This hypothesis is in line with a recent finding attributing to α-synuclein a significant role in cerebral inflammatory responses^20^ through its ability to modulate brain lipid metabolism. Equally important, it lends support to our theory correlating neuronal inflammation to mitochondrial dysfunction through NCX3 impairment. On the other hand, it has recently been demonstrated that α-synuclein is also localized at the mitochondrial level where it plays a key role in regulating Ca^2+^ homeostasis, mitochondrial depolarization, and dynamics^19,51^. By promoting dopaminergic neurons and fibers degeneration, these effects could explain the locomotor impairment observed in A53T mice, in line with data already described by Paumier et al^52^. Indeed, in the open field, results from spontaneous locomotor testing indicated that A53T mice traveled significantly greater distances than wild-type littermates at 6 and 12 months^52^. One possible explanation for this apparent discrepancy is that in our work the open field test was performed for a longer period of time (15 min) in comparison to the experiment performed by Paumier et al.^52^.

In conclusion, our findings demonstrate that changes in NCXs expression and activity in the midbrain and striatum of 12-month-old A53T mice contribute to the loss of dopaminergic neurons. Interestingly, our results suggest that an imbalance of the mechanisms involved in the regulation of cytosolic and mitochondrial Ca^2+^ homeostasis might be involved in the degeneration of dopaminergic neurons in the presence of a mutated form of α-synuclein A53T, thus revealing new potential players in the pathophysiology of PD.

## Materials and methods

### Transgenic mice bearing α-synuclein A53T human mutation

Twelve-month-old mice expressing the human A53T α-synuclein mutation under the control of a *prion* promoter (Pmp-SNCA*A53T)^53^ were obtained from The Jackson Laboratory. Mice hemizygous for the α-synuclein A53T mutation were bred on a mixed C57Bl/6 × C3H background to produce transgenic and non-transgenic littermates. To identify A53T mice, PCR was performed according to the protocol provided by The Jackson Laboratory. All mice were housed in groups of 1–5, in temperature and humidity-controlled rooms under a 12-h light/dark cycle and fed an ad libitum diet of standard mouse chow. Experiments were performed on male mice according to the international guidelines for animal research. Approved by the Animal Care Committee of “Federico II” University of Naples, Italy, they were in compliance with the Italian guidelines for care and use of experimental animals and with the European Communities Council Directive (2010/63/EEC).

### Primary midbrain neurons

Primary midbrain cultures were isolated from brains of 15-day-old A53T and WT mouse embryos, and prepared by modifying the previously described method proposed by Fath and collaborators^54^. The tissue was minced and incubated in a dissection medium containing MEM, NaHCO_3_, and dextrose for 30 min at 37 °C. After incubation, the suspension was centrifuged and subjected to mechanical dissection to obtain cell suspension. Then the cells were placed on poly-D-lysine-coated (100 µg/ml) plastic dishes, in MEM/F12 culture medium containing glucose, 5% deactivated fetal bovine serum, 5% horse serum, glutamine (2 mM), penicillin (50 U/ml), and streptomycin (50 μg/ml). For confocal experiments, the cells were plated on glass coverslips coated with poly-D-lysine^55^. The next day, cells were treated with cytosine-β−D-arabino-furanoside in vitro (10 μM) to prevent non-neuronal cell growth. Neurons were cultured at 37 °C in a humidified 5% CO_2_ atmosphere and used after 10 days in culture (DIV) for all the experiments described.

Primary mature neuronal culture (10 DIV) recapitulate the features of the adult brain as well as those of the disease-affected brain, thus representing an useful model to reproduce in vitro neurodegenerative diseases^56–63^.

### Western blot Analysis

Mice brain tissue and primary neurons were lysed in a buffer containing 20 mM Tris-HCl (pH 7.5); 10 mM NaF; 150 mM NaCl; 1 mM phenylmethylsulphonyl fluoride (PMSF); 1% NONIDET P-40; 1 mM Na_3_VO_4_; 0.1% aprotinin; 0.7 mg/ml pepstatin; and 1 μg/ml leupeptin. Homogenates were centrifuged at 14.000 rpm for 20 min at 4 °C. Supernatant was collected and used for protein content quantification using the Bradford method^48^. The total protein amount used for each sample was 50 μg. Proteins were separated on 8% sodium dodecyl sulfate polyacrylamide gels with 5% sodium dodecyl sulphate stacking gel (SDS-PAGE), and subsequently transferred to nitrocellulose membranes. The membranes were blocked in 5% non-fat dry milk in 0,1% Tween 20 (TBS-T; 2 mmol/l TrisHCl, 50 mmol/l NaCl, pH 7,5) for 1 h at room temperature and incubated overnight at 4 °C in the blocking buffer containing 1:1000 antibody for NCX1 (polyclonal rabbit antibody), 1:1000 antibody for NCX2 (polyclonal rabbit antibody), 1:5000 antibody for NCX3 (polyclonal rabbit antibody), 1:1000 antibody for α-synuclein (monoclonal rabbit antibody), and 1:10000 antibody for tyrosine hydroxylase (TH, monoclonal mouse antibody). Next, all membranes were washed three times with a solution containing Tween 20 (0,1%) and subsequently incubated with the secondary antibodies f`or 1 h (1:2000) at room temperature. The immunoreactive bands were visualized by enhanced chemiluminescence. The optical density of the bands was normalized with those of α-tubulin and measured by ImageJ program (U.S. National Institutes of Health, USA).

### Immunohistochemistry

A53T and WT mice were deeply anesthetized and sacrificed by transcardiac perfusion with 4% paraformaldehyde in phosphate buffer (PB, 0.1 M, pH 7.4). The brains were removed, post-fixed for 2 h, and processed for immunohistochemical studies as previously described^64^. a volume of 50-μm sections from the striatum and SNc (midbrain) of A53T and WT mice (striatum: from 1.34 to 0.74 mm; SNc: from −2.92 to −3.52 mm relative to bregma, according to the mouse brain atlas of Paxinos and Franklin^65^) were cut coronally on a vibratome and immunoreacted with TH, GFAP (mouse anti-GFAP, 1:400), and IBA-1 (polyclonal goat anti-IBA-1, 1:1000), as astroglial and microglial markers, respectively. Moreover, to investigate NCX1 and NCX3 levels in dopaminergic neurons and microglial cells, double immunostaining for IBA-1+NCX1, IBA-1+NCX3, TH+NCX1, and TH+NCX3 was performed in the striatum and SNc. For diaminobenzidine visualization of TH in the SNc, a biotinylated goat anti-mouse immunoglobulin G (IgG) (1:500) was used as a secondary antibody and the avidin–biotin–peroxidase complex protocol was followed^64^. For the visualization of TH in the striatum, GFAP and IBA-1 in the striatum and SNc, the proper fluorescent secondary antibody (AlexaFluor® 488-labeled donkey anti-mouse IgG for TH and GFAP; AlexaFluor® 594-labeled donkey anti-goat IgG for IBA-1, 1:400) was used. For the double immunostaining of IBA-1, TH, NCX1, and NCX3, AlexaFluor® 594-labeled donkey anti-goat IgG, AlexaFluor® 488-labeled donkey anti-mouse IgG, AlexaFluor® 488-labeled donkey anti-mouse IgG and AlexaFluor® 594-labeled donkey anti-rabbit IgG (1:400) were used as secondary antibodies. To allow visualization of cell nuclei in the fluorescent staining, sections were finally incubated for 10 min with 4′,6-diamidine-2′-phenylindole dihydrochloride (DAPI, 1:10,000). The sections were mounted on gelatin-coated slides, dehydrated, and coverslipped. Standard control experiments were performed by omission of the primary or secondary antibody, and yielded no cellular labeling.

*Stereological analysis of TH-positive neurons in SNc*. Stereological analysis of the total number and density of TH-positive neurons in the SNc and of the volume of the SNc was performed on both hemispheres, using a software (Stereologer) linked to a motorized stage on a light microscope^66^. The SNc region was outlined at low magnification (×2) and sampling of cells was achieved by using automatically randomized sampling and optical dissector (50 × 50 × 15 μm). Cells were sampled with a ×40 objective through a defined depth with a guard zone of 2 μm. Coefficient of error ranged from 0.05–0.1^66^.

*Analysis of TH-positive fibers in striatum*. Images of single wavelength were obtained with an epifluorescence microscope (Axio Scope A1) connected with a digital camera (1.4 MPixels, Infinity 3–1, Lumenera). In each of the three brain sections, two portions from striatum (dorsolateral and ventromedial), left and right, were acquired using a ×20 objective. The density of immunoreacted fibers was determined quantitatively using the ImageJ program. Sections were captured in black and white 8-bit monochrome and the density of fibers was determined in fixed regions using a threshold level that was kept constant across all images. The final values are expressed as a percentage of the WT group. No significant differences in the density of immunoreacted fibers were seen between the three sections. For each level of the striatum, the obtained value was first normalized with respect to WT, then values from different levels were averaged.

*Analysis of GFAP- and IBA-1-positive cells in striatum and SNc*. In each of the three brain section, two portions from the striatum (dorsolateral and ventromedial) and the whole SNc, left and right, were acquired with the same epifluorescence microscope cited above. Sections were captured at ×20 magnification for striatum analysis, or at ×10 magnification for SNc analysis. The number of cells for each level of the striatum and SNc, labeled with the nuclear marker DAPI, was counted manually using the ImageJ program. Cells were counted when a cell body from which processes extended was observed, or when the processes were all directed toward a central point that corresponds with the likely position of the cell body deeper in the tissue^67^. GFAP-/IBA-1-expressing fibers without a clear indication of the associated cell body were not counted. To determine whether the quantification of the number of GFAP-/IBA-1-positive cells in a single section accurately reflects the total number of GFAP-/IBA-1-positive cells, we analyzed only cells labeled with the nuclear marker DAPI.

*Analysis of IBA-1+NCX1, IBA-1+NCX3, TH+NCX1, and TH+NCX3 in striatum and SNc*. Each of the three brain section, i.e., the dorsolateral and ventromedial striatum, and the whole SNc, left and right, were acquired using a ×40 objective from the same epifluorescence microscope cited above. Quantitative analysis of colocalization of IBA-1 and NCX1 or NCX3, and TH and NCX1 or NCX3 was conducted using *ImageJ* plug-in JACoP (Just Another Colocalization Plugin)^68,69^. A correlation of signal intensity was calculated as a Pearson correlation coefficient (Rr). Rr is a quantitative measurement that estimates the degree of overlap between the fluorescence signals obtained from two channels^69^.

### Mitochondrial calcium concentrations [Ca^2+^]_m_ and cytosolic calcium concentrations [Ca^2+^]_c_

Neurons obtained from A53T and WT mouse embryos were loaded with X-Rhod-1 (0.2 μM) for 15 min in a medium containing 156 mM NaCl, 3 mM KCl, 2 mM MgSO_4_, 1.25 mM KH_2_PO_4_, 2 mM CaCl_2_, 10 mM glucose, and 10 mM Hepes (pH 7.35). At the end of the incubation period, cells were washed three times in the same medium. An increase in mitochondria-localized intensity of fluorescence was indicative of mitochondria Ca^2+^ overload^27^.

[Ca^2+^]_c_ was measured using the fluorescent dye Fluo-3AM acetoxymethyl ester (Fluo-3AM).

Cells were loaded with Fluo-3AM (5 μM) for 30 min at room temperature in the same medium described above. At the end of incubation, cells were washed three times in the same medium. An increase in [Ca^2+^]_c_ intensity of fluorescence was indicative of cytosolic Ca^2+^ overload^70^. The advantage to use fluo3 was that this calcium indicator can be loaded into the cells together with the mitochondrial calcium indicator X-Rhod-1, thus allowing a simultaneous comparison of calcium levels in the cytoplasmic and mitochondrial compartment.

Confocal images were obtained using Zeiss inverted 700 confocal laser scanning microscopy and a ×63 oil immersion objective. The illumination intensity of 543 Xenon laser used to excite X-Rhod-1, and of 488 Argon laser used to excite Fluo-3AM fluorescence, was kept to a minimum of 0.5% of laser output to avoid phototoxicity.

### Evaluation of motor activity

*Open Field Test*. Spontaneous exploratory locomotor behavior was evaluated by means of the open field test. The open field test provides a highly efficient paradigm for phenotype characterization of A53T mice^71^. Indeed, the test has been successfully used with other familial PD mouse models^72^. Behavioral activity was evaluated in 12-month-old A53T and WT mice.

The Open Field apparatus, that is a Plexiglas square arena (45 × 45 cm, 40 cm high), was placed in a homogenously lit experimental room. For each test, mice were placed individually in the center of the square and allowed to explore it for 15 min^73^. Fifteen minutes is a long enough time to evaluate the impairment in locomotor activity once the mice have become familial to the new environment.

Total traveled distance was measured with a video-tracking software.

*Pole Test*. The pole test assesses the agility of animals and was first designed for mice by Ogawa and colleagues to measure bradykinesia^74^. This task, involving skilled fore-limb grasping and maneuvering, requires an intact basal ganglia and activation of the rubrospinal pathway. This task, therefore, is very sensitive to nigrostriatal dysfunction^74–78^.

Mice were placed head upward at the top of a vertical rough-surfaced pole (diameter 1 cm; height 55 cm) and a recording of the amount of time spent by the mouse to reach the floor was performed. The performance was scored until the mouse reached the floor. For each experimental section, animals received three trials and the average scorers were expressed in seconds.

*Beam Walking test*. The motor performance and coordination of A53T and WT mice were evaluated with the beam walking test^77,79,80^. In this test, mice were trained to traverse the length of a plexiglas beam divided into four sections (25 cm each, 1 m total length). Each section of the beam had a different width: 1, 2, 3, and 4 cm; the beam, placed on a table, led directly into the home cage. Mice received two days of training before testing. On the first day, mice received two assisted trials, involving the placement of the mouse on one extremity of the beam with the home cage in close proximity to the animal. This encourages forward movement along the beam. After two assisted trials, mice were able to traverse the entire length of the beam unassisted. The two-day training sessions ended when all mice completed five unassisted runs across the entire length of the beam. To render the task even more challenging on the day of the test, a mesh grid (1-cm squares) of corresponding width was placed over the beam surface. Mice were videotaped for a total of five trials. An error was counted when, during a forward movement, a limb slipped through the grid; therefore every mice could make a maximum of four slips per step. By scoring each limb slip individually, the severity of the error could be measured. Time to traverse, number of steps, and error per step scores were calculated across all the five trials and averaged for each group.

### Statistical analysis

Data were collected from a minimum of three independent experimental sessions for in vitro studies, and from eight animals per group for in vivo experiments. Ca^2+^ measurements were performed at least in 20 cells for each of the three independent experimental sessions. Data were expressed as mean ± S.E.M. Statistical comparisons between A53T and WT mice, cells and their respective controls were performed using the one-way ANOVA test, followed by Newman Keul’s test. *P* value < 0.05 was considered statistically significant.

## Electronic supplementary material

S1

S2

S3

S4

Supplementary Figure Legends
