# Supplementary figures and images for: NCX1 and NCX3 as potential factors contributing to neurodegeneration and neuroinflammation in the A53T transgenic mouse model of Parkinson’s Disease
Source: Cell Death Dis. 2018 Jun 25;9(7):725. doi: 10.1038/s41419-018-0775-7 (PMC6018508; doi:10.1038/s41419-018-0775-7)

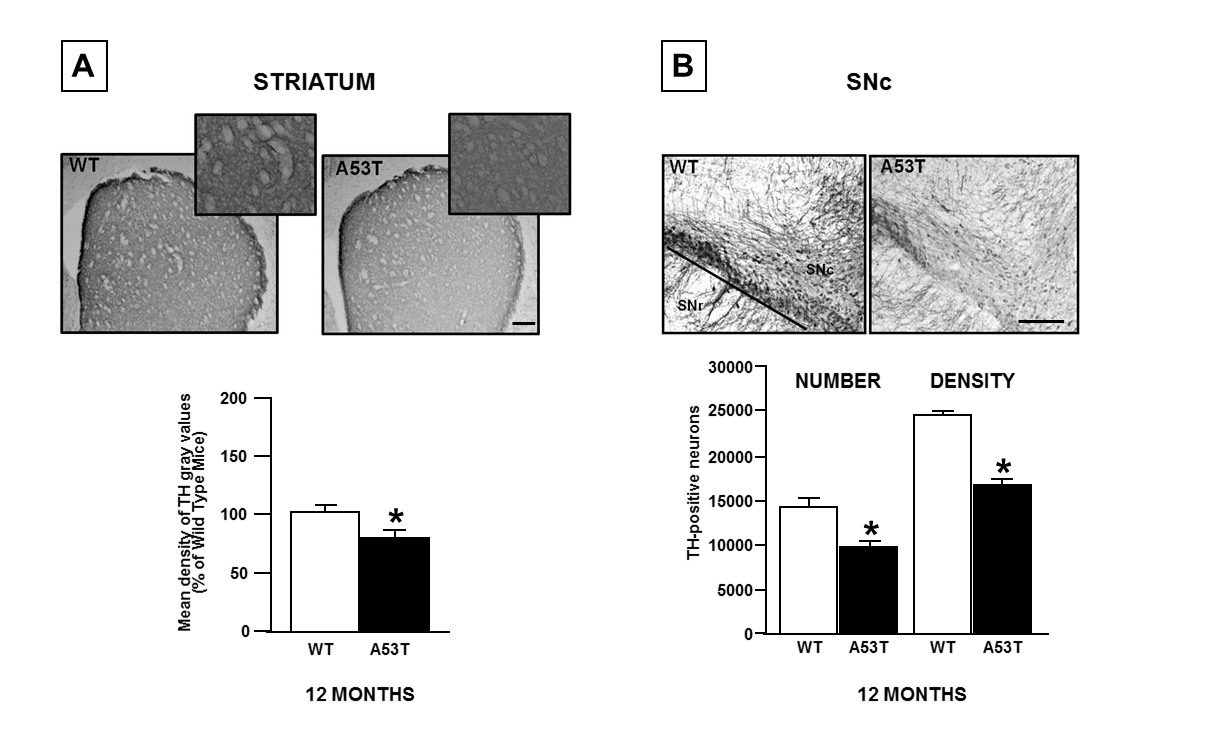

Supplement: Supplementary file 1 — S1 [file 41419_2018_775_MOESM1_ESM.tif]

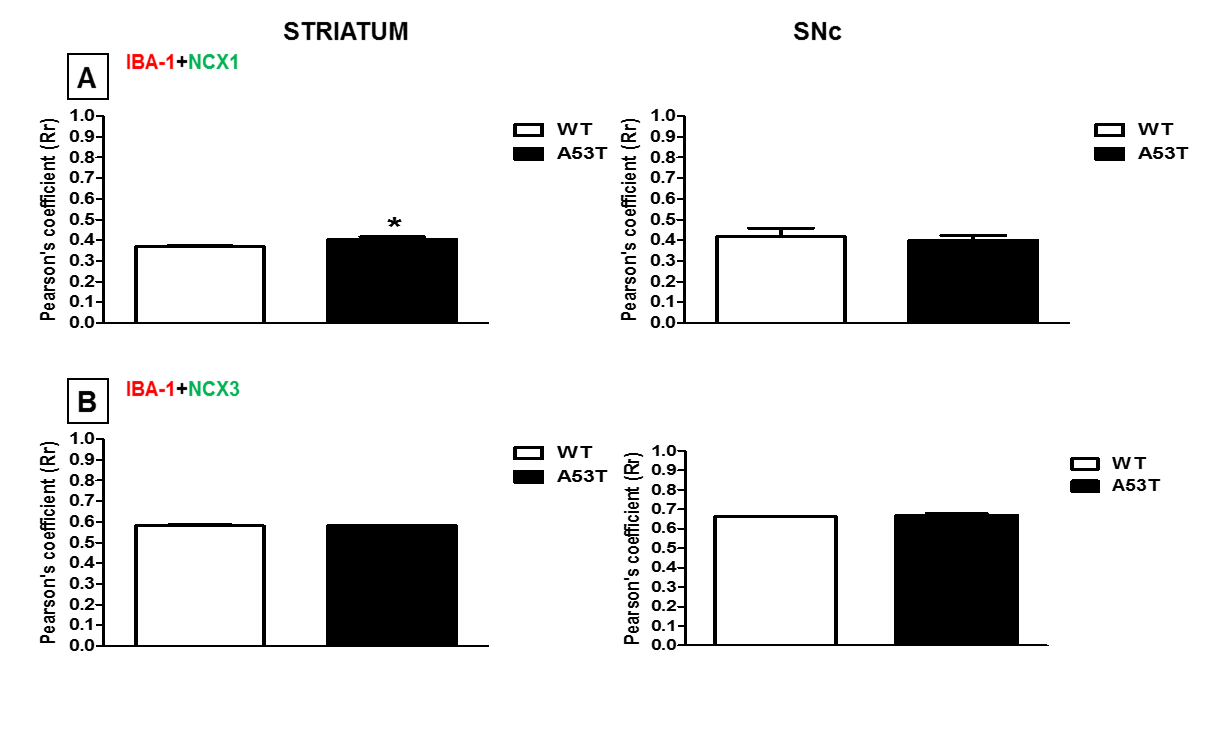

Supplement: Supplementary file 2 — S2 [file 41419_2018_775_MOESM2_ESM.tif]

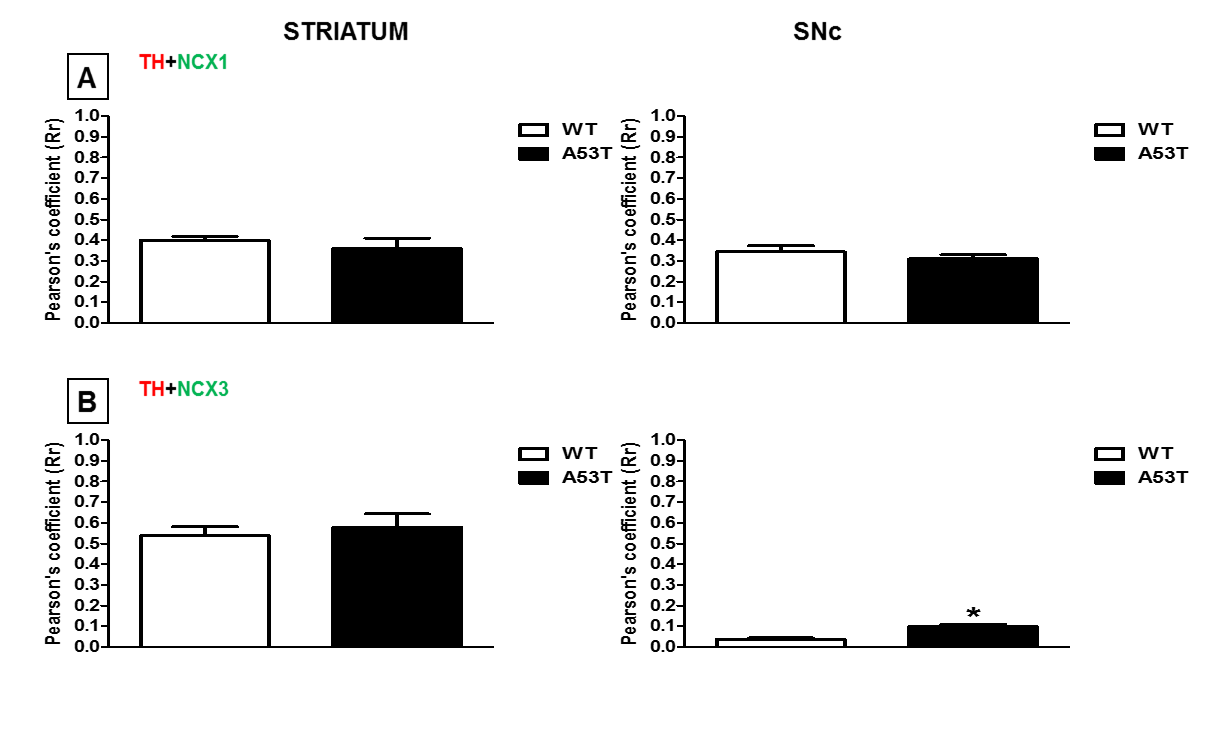

Supplement: Supplementary file 3 — S3 [file 41419_2018_775_MOESM3_ESM.tif]

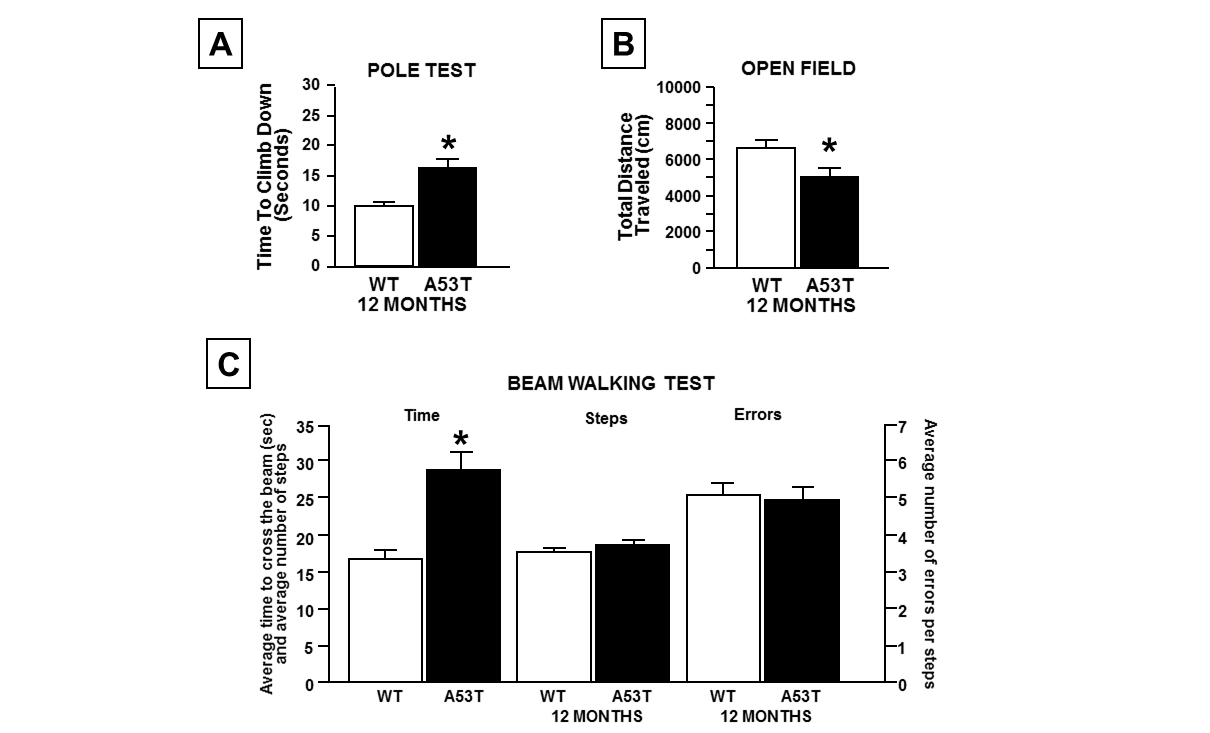

Supplement: Supplementary file 4 — S4 [file 41419_2018_775_MOESM4_ESM.tif]
