# Supplementary material for: NCX1 and NCX3 as potential factors contributing to neurodegeneration and neuroinflammation in the A53T transgenic mouse model of Parkinson’s Disease
Source: Cell Death Dis. 2018 Jun 25;9(7):725. doi: 10.1038/s41419-018-0775-7 (PMC6018508; doi:10.1038/s41419-018-0775-7)
Supplement: Supplementary file 5 — Supplementary Figure Legends [file 41419_2018_775_MOESM5_ESM.docx]

Supplemental Material

**Figure S1. TH expression in striatum and substantia nigra *pars compacta (*SNc) of A53T and WT mice.** Representative sections and histograms of the striatum **(A)** and SNc **(B)** immunostained for TH. For TH in striatum, values are expressed as mean ± S.E.M of the percentage of WT mice, whereas for TH in SNc values are expressed as mean ± S.E.M. N=8 animals were used for each experimental group. **P*<0.001 compared with WT mice. Scale bar: 50 µm.

**Figure S2. Quantitative analysis of IBA-1 and NCX1 or NCX3 colocalization in A53T and WT mice: (A)** Bar graphs representing the mean ± S.E.M. of the Pearson’s coefficient (Rr) in striatum (on the left) and SNc (on the right) for IBA-1+NCX1 immunoreactivity. **(B)** Bar graphs representing the mean ± S.E.M. of the Pearson’s coefficient (Rr) in striatum (on the left) and SNc (on the right) for IBA-1+NCX3 immunoreactivity. N=8 animals were used for each experimental group. **P*<0.05 vs respective WT mice.

**Figure S3. Quantitative analysis of TH and NCX1 or NCX3 colocalization in A53T and WT mice: (A)** Bar graphs representing the mean ± S.E.M. of the Pearson’s coefficient (Rr) in striatum (on the left) and SNc (on the right) for TH+NCX1 immunoreactivity. **(B)** Bar graphs representing the mean ± S.E.M. of the Pearson’s coefficient (Rr) in striatum (on the left) and SNc (on the right) for TH+NCX3 immunoreactivity. N=8 animals were used for each experimental group. **P*<0.05 vs respective WT mice.

**Figure S4. Motor performance in A53T and WT mice monitored by: (A)** Pole test: difference of the time spent to climb down from the pole in A53T and WT mice reported as mean ± S.E.M. **(B)** Open Field test: values of the distance travelled expressed in cm and reported as mean ± S.E.M. **(C)** Beam Walking test: time to traverse, number of steps and errors per step scores calculated in A53T and WT mice reported as mean ± S.E.M. across all five trials and averaged for each group. N=8 animals were used for each experimental group. **P*<0.05 vs respective WT mice.
